# Mapping public health responses with attitude networks: the emergence of opinion‐based groups in the UK’s early COVID‐19 response phase

**DOI:** 10.1111/bjso.12396

**Published:** 2020-07-04

**Authors:** Paul J. Maher, Pádraig MacCarron, Michael Quayle

**Affiliations:** ^1^ Department of Psychology Centre for Social Issues Research University of Limerick Ireland; ^2^ MACSI (Mathematics Applications Consortium for Science and Industry) Department of Mathematics & Statistics University of Limerick Ireland; ^3^ Department of Psychology School of Applied Human Sciences University of KwaZulu‐Natal Scottsville South Africa

**Keywords:** opinion‐based groups, identity, attitude networks, COVID‐19, political polarization

## Abstract

Partisan patterns of compliance with public health measures are a feature of early COVID‐19 responses. In many cases, these differences in behaviour relate to pre‐existing group identities. However, in times of rapid societal change, novel opinion‐based groups can emerge and provide a new basis for partisan identification and divergent collective behaviour. Here, we use network methods to map the emergence of opposing opinion‐based groups and assess their implications for public health behaviour. In a longitudinal study, we tracked public health attitudes and self‐reported behaviour in a sample of UK participants over four time points. Network visualisation reveal a rift in attitudinal alignment over time and the genesis of two distinct groups characterised by trust, or distrust, in science (Study 1a; *N *= 253). These groups also diverge in public health behaviour. In a brief follow‐up study (*N *= 206), we find that this opinion polarization partially reflects underlying societal divides. We discuss implications for opinion‐based group research and public health campaigns.

## Background

Effective societal responses to severe acute respiratory syndrome coronavirus 2 (SARS‐CoV‐2) and the associated infectious disease (COVID‐19) pandemic require long‐term and large‐scale trust among disparate groups in society (Vaughan & Tinker, 2009). Worryingly, there is reason to believe that partisan compliance is a feature of COVID‐19 public health responses (e.g., Allcott *et al*., 2020; Van Bavel *et al*., 2020). Even as restrictions ease, there is a danger of subsequent waves of infections if public health messaging cannot engender solidarity (Haslam *et al*., 2018). Tracking the emergence of partisan rifts in public health attitudes allows social psychologists to map identity‐based factions in public health behaviour.

Social influence is an important moderator of public health messaging. For example, communications implicitly convey group norms (Nightingale, Quayle & Muldoon, 2017) and this can lead to misperceived health risks (Berkowitz, 2004) and divergent health behaviour (Jetten *et al*., 2014). In the United States, attitudes and behavioural responses towards COVID‐19 rapidly diverged on political party lines (Wise *et al.*, 2020), with democrats more likely than republicans to report vigilant handwashing and the avoidance of large crowds (YouGov, 2020). Indeed, there is evidence suggest a partisan rifts in health behavior, even after controlling for alternative explanations (Allcott *et al*, 2020; Gollwitzer *et al*. 2020)^1^Please note, these papers are pre‐prints and have not been peer‐reviewed..

Partisan divisions in health attitudes and behaviours are detectable in the United States as they fall relatively cleanly along political divides. However, as previously seen during the Brexit debate in the United Kingdom, new ‘opinion‐based groups’ (McGarty, Bliuc, Thomas, & Bongiorno, 2009) can emerge from social processes without clear relations to prior groups or socio‐political structures. Even in the US, the presence of ideological structure among public attitudes is often overemphasised (Converse, 1964). Therefore, it is important to be able to track the dynamic emergence of any partisan rifts in public health attitudes without using preconceived categories or retrospective inference. For clarity, we describe these emergent opinion‐based structures as *factional* to emphasize that they offer a new basis for identity alignment, as opposed to *partisan* structures that align with pre‐existing socio‐political identities. In this paper, we track public health attitudes in a sample of UK participants during the early stages of the COVID‐19 pandemic. Using a novel network‐based method, we explore whether opposing attitude‐based clusters emerge over time and investigate whether factional attitude alignment becomes a basis for divergence in public health behaviour.

### Opinion‐based groups

Attitudinal overlap can be the basis for perceived similarity and social group formation (Macy, Deri, Ruch, & Tong, 2019), as observed in opinion‐based groups (Bliuc *et al*., 2015). Opinion‐based groups are groups formed around shared opinions. Importantly, these groups can form rapidly through online interaction (Garcia, Galaz, & Daume, 2019) and foster forms of identification that transgress more categorical group boundaries. Here, a single topic can become a nexus for social identification (Bliuc *et al*., 2007) and intergroup conflict (Bliuc *et al*, 2015). For example, climate change ‘sceptics’ and ‘believers’ have distinct social identities based around global warming attitudes (Bliuc *et al*., 2015). A further defining feature of groups formed around shared attitudes is the ease with which they facilitate the coordination of behaviour.

Shared attitudes are a basis for collective identity and agency (McGarty, *et al*., 2009), since once you know what you stand for it is easy to agree on how to act. Consensus on health‐related attitudes can influence health behaviour (Montoya‐Williams & Fuentes‐Afflick, 2019) and facilitate coordinated online activity (Garcia *et al*., 2019). For example, Garcia *et al*. (2019) tracked the rapid formation of an online community of Twitter users connected through their disapproval of non‐meat diets. In anticipation of an upcoming Lancet report highlighting the science behind healthy and sustainable eating, the community coordinated the proliferation of #yes2meat as a means to effectively dominate coverage of the launch on Twitter. Indeed, many forms of health communication are susceptible to misinformation and partisan persuasion (Broniatowski *et al*., 2018). We already see motivated partisan persuasion by disparate groups in the COVID‐19 pandemic (e.g., protests against lockdown), and this can undermine the solidarity required for public health compliance.

### Polarization and attitude networks

In times of social crises, attitudes rapidly coordinate and polarize (Smith *et al*., 2019) as people seek clarity from leaders and similar others (Kruglanski *et al*., 2006; Mueller, 1970). Thus, during the Brexit process, previously innocuous opinions like one’s view of the EU became a catalyst for long‐term realignments in British politics (Hobolt, Leeper, & Tilley, 2020). Similarly, economic and political attitudes polarized in the wake of the Great Recession (McCarty, Poole, & Rosenthal, 2016) and the election of Donald Trump (Maher, Igou, & Van Tilburg, 2018). This attitude polarization may build upon pre‐existing rifts (e.g., political divides) but it is often not reducible to political or demographic categorizations (McGarty, *et al*., 2009). COVID‐19 has spurred societal change at an alarming rate, and this too will shift the structure of attitudes in society.

Network methods reveal how even small changes can lead to rapid shifts in otherwise stable attitudinal relationships (Dalege *et al*., 2016). Assessing the connection between attitudes in a network helps explain the central role of identity in coordinating beliefs and behaviour (Brandt, Sibley, & Osborne, 2019). Importantly, attitudes propagate through group structures (Jost, Ledgerwood, & Hardin, 2008) and can quickly coordinate into factional alignment. We propose that networks of attitude agreement simultaneously produce symbolic structures and group structures that bind people together (MacCarron *et al*., 2020; Quayle, 2020) and that social crises (e.g., pandemics) accelerate this process. In two complimentary studies, we investigate (i) the emergence of factional alignment in health attitudes during the early phase of the COVID‐19 pandemic, (ii) consequences for maintaining public health behaviour, and (iii) the contribution of pre‐existing social categories.

## Study 1a

In the United Kingdom, public trust in health officials is high and typically not a partisan issue (Wellcome Global Monitor, 2018). However, in times of crisis, novel attitude coordination can occur as people seek clarity and certainty (e.g., Mueller, 1970). This study aims to (i) investigate whether emerging factions can be detected in public health attitude coordination and (ii) assess how this corresponds to public health behaviour.

### Methods

#### Participants

Based on a preliminary network analysis of representative UK data with the same items (Wellcome Global Monitor, 2018), we estimated that at least 200 participants would be required to visualize opinion‐based groups. To accommodate longitudinal attrition, we aimed for 300 participants at Time 1 (T1). Participation was restricted to UK residents recruited online through Prolific Academic (Prolific.ac) and paid £0.75 per time point.

We planned three waves of data collection to coincide with significant events in the UK Government response to COVID‐19. We collected T1 data on 9 March, three days after the first reported fatality in the United Kingdom. We excluded three participants for failing an attention check, leaving 297 (239 women; *M*_age_ = 34.73, *SD* = 11.16). We collected Time 2 (T2) data from the same participants a week later (16 March; *N *= 286 participants), three days after UK risk level was raised to high. We removed five for failing an attention check and nine others could not be matched to T1. This left 272 (215 women; *M*_age_ = 34.80, *SD* = 11.21).

Time 3 (T3) data were collected a week later (23 March), three days after the closure of non‐essential business and the ban on ‘non‐essential’ travel in the United Kingdom (*N *= 253). Two participants were removed for failing attention checks and a further 16 could not be matched to both T1 and T2. This left a final sample of 235 (184 women; *M*_age_ = 35.60, *SD* = 11.41) participants for analysis across all three time points.

#### Materials and procedure

Online supplementary materials contain all items answered by participants (https://osf.io/a9hdn/?view_only=ee44ced8b3ed4ca0824f0150f60b60b4). We measured public health attitudes with 11 items from the Wellcome Trust health survey (Wellcome Trust Global Monitor, 2019). These assessed participants’ trust in: science, scientists, the government, doctors, journalists, charity workers, traditional healers, community, and vaccines.

We measured compliance with public health advice with three items relating to physical distancing and handwashing which formed one compliance scale (T1 α = .70; T2 α = .74; T3 α = .73)^2^The study survey also included other measures that were unrelated to the current studies aims. Two behavioural measures were no longer relevant after lockdown was announced. These are included in supplementary materials..

Finally, three items assessed epistemic clarity. We asked participants how well they understood COVID‐19 precautions, how much they made sense and how meaningful they were (T1 α = .79; T2 α = .76; T3 α = .75). Participants responded using 7‐point scales (1 = not at all; 7 = a great deal).

### Results

#### Analytical approach

**Figure 1:**
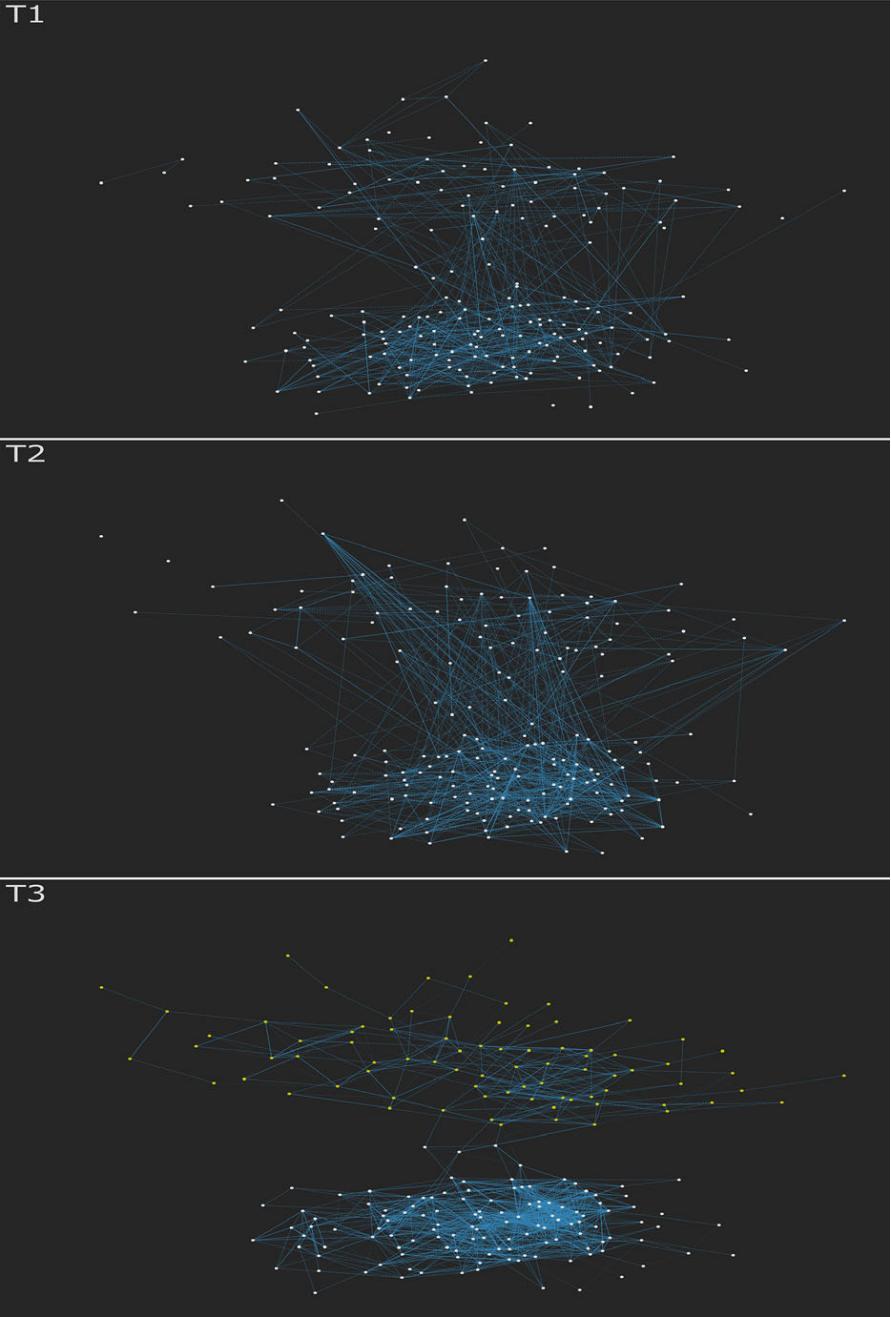
The participant projection of the survey at each time point. Blue edges represent two participants who agree on many attitudes. Yellow nodes at T3 represent the sceptics cluster. [Colour figure can be viewed at wileyonlinelibrary.com]

First, a bipartite graph (i.e., network) of public health attitudes was constructed for each survey time point. This is a graph with two types of nodes, where edges can only connect nodes of different types. The bipartite graph can be projected either to show how people are linked by shared attitudes or attitudes are linked by the people who share them. In Figure 1, we show the participant projection at the three different time points. Here, a link represents the proportion of attitudes shared by two participants. In time 3, we observe two clusters that are linked by a total of four edges (see supplementary materials for further details).

**Figure 2:**
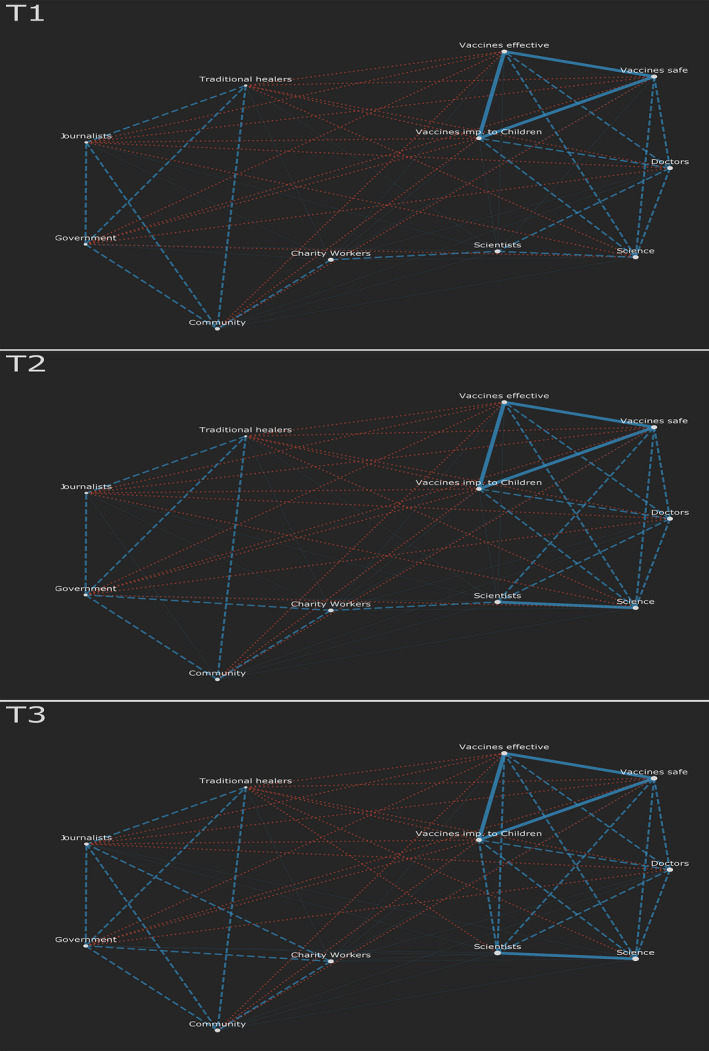
The attitude projection at each time point. Blue edges represent agreement and red disagreement. The weight of an edge corresponds to the number of participants sharing similar response to those items. In T3, there are no strong blue edges connecting the lower and upper clusters. [Colour figure can be viewed at wileyonlinelibrary.com]

A similar method is used for the attitude projection in Figure 2. Here, the edges represent the number of people sharing these attitudes. If the edge is blue, most participants align on these attitudes, if red there is mostly disagreement. For example, many participants who trust doctors distrust government and vice versa. Please see online supplementary materials for more details.

#### Group genesis

Figures 1 and 2 reflect a specific form of consensus‐based polarization, not evident in conventional mean‐based comparisons. K‐means clustering confirms the two distinct groups evident in attitude networks at T3, and a chi‐square independence test confirmed the correspondence of these groupings with those identifiable in the figures above, *X^2^*(1) = 186.31, p < .001. There is a larger cluster of white nodes (the science‐trusting cluster; *N* = 141) and a smaller cluster of yellow nodes (the science‐sceptic cluster; *N* = 87) at T3. These emerged as consensus built within distinct groupings and differences built between them. Over time, the number of people combining positive attitudes towards science with positive attitudes towards government and charity becomes smaller (evidenced by the reduction in edges across these components).

We verified the clustering evident above by assessing individual‐level attitude change between participants in each cluster in a repeated measures MANOVA with time varying within‐subjects, clusters between‐subjects, and attitudes towards doctors, science, scientists, and vaccines as multiple DVs. We found a significant multivariate effect of time (*F*[12, 206] = 3.08, *p *< .001, η^2^ = .15, Λ = .85), cluster (*F*[6, 212] = 71.08, *p *< .001, η^2^ = .67, Λ = .33) and a time x cluster interaction (*F*[12, 206] = 7.55, *p *< .001, η^2^ = .30, Λ = .69). Specifically, attitudes towards scientists and doctors differed significantly across time, with trust in scientists growing from T1 (*M* = 2.87, *SD* = 1.01), to T2 (*M* = 2.95, *SD* = 1.01) to T3 (*M* = 3.12, *SD* = 1.00), *F*(2, 434) = 3.49, *p* = .031, η2 = .02 and trust in doctors growing from T1 (*M* = 3.28, *SD* = 1.00), to T2 (*M* = 3.39, *SD* = .92), to T3 (*M* = 3.49, *SD* = .88), *F*(2, 434) = 4.92, *p* = .008, η2 = .02.

**Figure 3:**
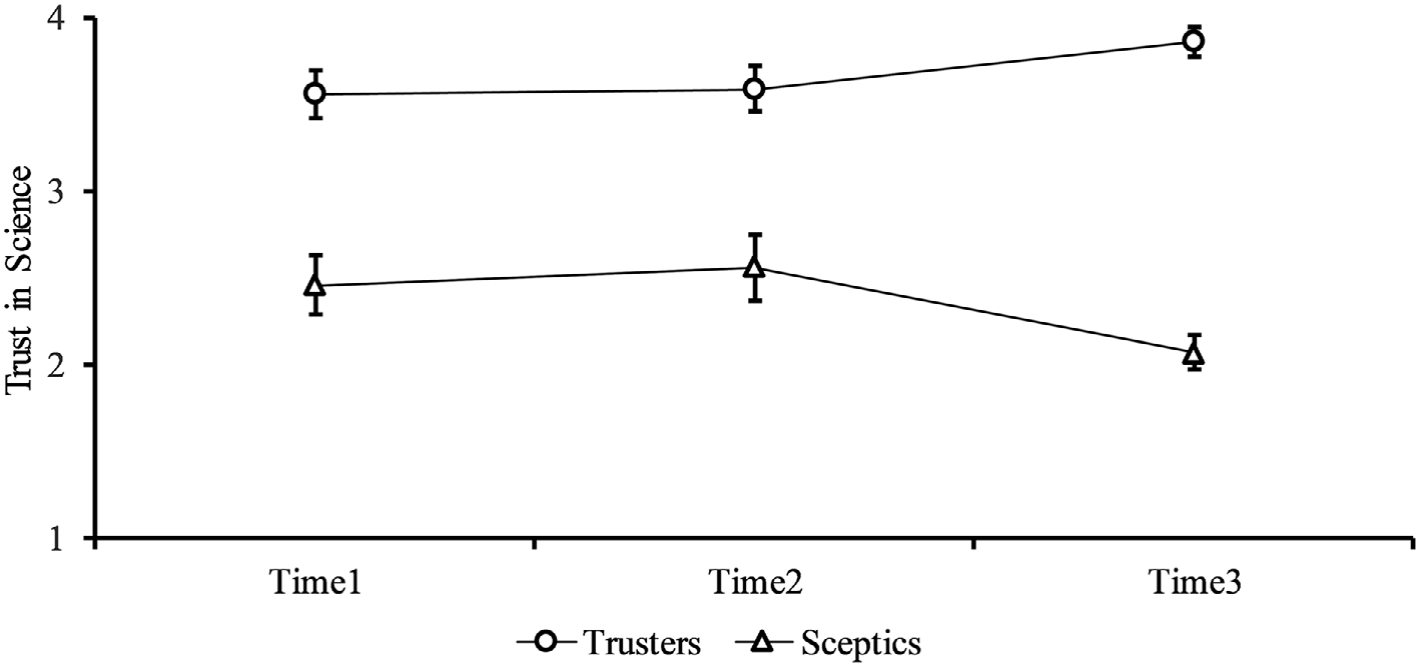
Mean levels of trust in science (1 = not at all; 4 = a lot) within each opinion‐based group across all time points. Error bars represent 95% confidence intervals.

However, these averages mask the time x cluster interaction, and univariate analysis reveals this is predominantly driven by a divergence in attitudes towards science, *F*(2, 434) = 23.95, *p *< .001, η^2^ = .10 and scientists, *F*(2, 434) = 21.50, *p *< .001, η^2^ = .09. Trust in science progressively decreased among those in the smaller cluster and increased among those in the larger cluster (see Figure 3). Hence, we refer to these clusters as science‐trusters and science‐sceptics, although we note that the network approach reveals attitudinal combinations not evident from analysing these variables independently. Importantly, this system‐level polarization would not have been evident without the network visualisation.

#### Behavioural compliance and epistemic clarity

We investigated whether these T3 clusters reflect opinion‐based groups by assessing differences in behaviour compliance and epistemic clarity at T3. Participants in the science‐sceptic cluster reported significantly lower behavioural compliance at T3 (*M* = 5.28, *SD* = 1.46) compared to those in the science‐truster cluster (*M* = 5.70, *SD* = 1.21), *F*(1,226) = 5.50, *p* = .020, η^2^ = .024, 90% CI [0.002, 0.065] and significantly lower epistemic clarity (*M* = 6.44, *SD* = 0.74) than the more trusting group (*M* = 6.68, *SD* = 0.72), *F*(1,226) = 5.94, *p *= .016, η^2^ = .026, 90% CI [0.003, 0.068]. Overall, attitude‐network analysis has enabled us to identify novel groups, for which public health messaging appears to have divergent effects.

## Study 1b

Study 1b explores whether the factional attitude alignment in Study 1a builds upon existing social divides and whether the attitudinal clusters we identify reveal something that was less evident from other means of Categorisation.

### Methods

#### Participants

We followed up Study 1a participants at a 4^th^ time point (T4) on April 6^th^, two weeks after T3 participation. Altogether, 261 participants took part, three failed attention checks and a further 41 could not be matched to participants who took part in all three previous time points. This left us with a total sample of 217 (171 women; *M*_age_ = 36.06, *SD* = 11.52).

#### Measures

We assessed a range of political and socio‐economic demographic variables.

##### Political measures

We measured how people voted in the 2016 Brexit referendum, as well as their views on Brexit on a 1‐7 scale (1 = strong remain; 7 = strong leave). We also assessed political orientation (1 = left wing; 7 = right wing) and 2016 general election vote.

##### Socio‐economic measures

We measured annual income level (1 = less than £10,400; 7 = more than £104,000), educational level (1 = primary education; 7 = doctoral degree), area of residence (urban v rural v suburban), and perceived social status (Adler *et al.*, 2000).

### Results

#### Group genesis

We assessed how people in attitude‐based clusters established at Study 1a differed in Brexit views, income, education, political orientation, and perceived social status. Clusters differed significantly in these five measures (see Table 1). Participants in the science‐sceptic cluster favoured leave over remain, reported a lower average income, lower average levels of education, and lower perceived social status. Chi‐square independence analyses tested associations between cluster and Brexit, general election 2019 vote, or residential area. Only Brexit vote was significantly related, *X^2^* (2) = 7.12, *p* = .029. Although there was a higher than expected portion of leave voters in the sceptics cluster (*n* = 30; from 81), there was also a substantial number in the science‐truster cluster (*n* = 27; from 129). Furthermore, there were a number of non‐voters in the sample (*n* = 39). Neither general election vote (*p* = .088) nor residential area (*p* = .237) was significantly related to attitude clusters.

**Table 1:** Differing demographics and political views across attitude‐based clusters

	Trusters	Sceptics	*F*	η^2^	*p*
	Means (SDs)				
Income level	4.13 (1.62)	3.50 (1.52)	7.99	.037	.005
Perceived Status	5.47 (1.46)	5.04 (1.39)	4.48	.021	.035
Education level	4.50 (1.24)	3.90 (1.25)	11.39	.052	.001
Brexit view *(1* = *remain; 7* = *leave)*	2.51 (1.90)	3.44 (2.21)	10.47	.048	.001
Political orientation *(1 = left; 7 = right)*	3.14 (1.39)	3.57 (1.33)	4.92	.023	.028

We assessed the contribution of each of these demographic factors in a multiple binary logistic regression. Specifically, we examined whether the probability of a participant belonging to either attitude‐based cluster was related to income, Brexit view, education, political orientation, or perceived SES. The model significantly predicted group assignment, *X^2^* (4) = 21.78, *p* < .001, with 67.6% of cases accurately classified. Brexit view was a marginally significant predictor, *B* = .164, *SE* = .086, *p* = .057, *OR* = 1.18, 95% CI [ 0.99,1.40] and the other variables had a non‐significant unique effect.

#### Health behaviour

There were no significant correlations between levels of behavioural compliance at T3 and Brexit view (*r* = −.046, *p* = .501), education levels (*r* = .013, *p* = .849), income levels (*r* = .041, *p* = .755), or perceived social status (*r* = .074, *p* = .275).

Overall, this analysis suggests that these emerging attitude‐based factions relate to existing rifts in society, both political and socio‐economic. However, these groups cannot be reduced to any one category while still capturing divides in COVID‐19 behaviour.

## Discussion

A bipartite network visualisation revealed factional attitudinal alignment emerging over time among UK participants sampled over a crucial 3‐week period of the COVID‐19 pandemic. This method provides a straightforward and theoretically informed way to conceptualize and inductively identify opinion‐based groups. These distinct attitude‐based factions differed in behavioural compliance, suggesting that trust in science and health officials is a core basis for emerging COVID‐19 opinion‐based groups.

The observed factions partially reflect underlying societal divides, such as income and educational disparities. However, these do not explain discrepancies in health behaviour. Instead, we observed a rapid emergence of factional consensus that did not obviously correspond to pre‐existing identity frameworks. Similar identity dynamics emerged around the ‘Brexit’ referendum, when opinion‐based groups rapidly coalesced across party lines, disrupting a relatively stable political system (Hobolt, Leeper, & Tilley, 2020). In contrast, other countries have seen health beliefs and behaviour coalesce along pre‐existing partisan lines (e.g., Allcott *et al*., 2020).

Practically, our results suggest directions for tailoring public health messages to maximize behavioural adherence (Hunecke, *et al*., 2010) and avoid factional divergence. Our analysis reveals an emerging basis for partisanship organized around trust or distrust of scientists and doctors. Using bipartite attitude alignment to identity distinct clusters, we observe factional differences in behavioural compliance and clarity around the reasons for restriction. In other words, an identifiable group of people are not getting (or accepting) the message. We expect that the increasingly polarized opinion ecosystem makes it more likely that factions will respond differently to health messaging.

Our visualisation of the evolution of factions suggests that stakeholders should focus somewhat on rebuilding understanding of science, including the notion that scientists will often be wrong before they are right and that disagreements are a natural part of the scientific process. This may be especially important in the future when vaccine uptake may be a crucial factor in defeating the virus. In general, (at the time of data collection) people in the United Kingdom trusted their communities and trusted vaccines. Future research may explore the effectiveness of messages that emphasize how scientists are members of our community and how vaccine development helps them to protect our communities.

Importantly, although our T3 networks demonstrate schism, our T1 networks show a strong overlap in public health attitudes. We note that these processes are dynamic and our strongest practical recommendation is for opinion networks to be tracked over time before, during, and after health behaviour campaigns to assess the possible emergence of opinion‐based groups that may undermine messaging or require different strategies.

Theoretically, we wish to make three points. First, shared attitudes are building blocks of identity (Quayle, 2020). Even attitudes about public health can quickly coalesce into opinion‐based factions. These rapidly emerging coalitions can become the basis for new emergent partisan identities. Previous research suggests that identities can develop when people are motivated to communicate their attitudes towards social change, because they encounter a situation that contradicts their view of how the world should be (Smith, Thomas & McGarty, 2015). In the present study, it is easy to imagine how the rifts that opened in the public health opinion space in the early weeks of the COVID‐19 crisis might be co‐opted by politicians, the media, or other agents in service of their identity entrepreneurship and political ambitions in the months and years to come (Reicher, Haslam, & Hopkins, 2005).

Second, the coordination of opinions can become a basis for the coordination of action (McGarty *et al*., 2009; Smith, *et al*., 2015) as we have observed in the United States where republican dissatisfaction with lockdown has been expressed in dangerous public protests. This is not to say that the same would inevitably happen in the United Kingdom; but rather that the emergent factional structures in the opinion space provide a starting point for such a social process to gather momentum.

Third, partisan polarization does not require extremism (Fiorina & Abrams, 2008), which is why we refer to polarization as attitude coordination. The factional opinion structures identified in the present analysis are not easily detected with conventional linear methods, but are evident in the bipartite network visualisation and cluster analysis. This work further demonstrated the benefits of network analysis for understanding dynamic social psychological phenomenon (see Abelson, 1967; Brandt *et al*., 2019; Dalege *et al*., 2016).

### Limitations

We have only limited evidence that the clusters we identify exist as psychological groups (Turner, 1982). Given the emergent nature of this phenomenon, it was not possible for us to measure group identification ahead of time. Rather, our research aims to track the emergence of a novel identity space (Quayle, 2020) based upon an increasing alignment of shared public health attitudes. We assert that opposing clusters of shared opinions foster a readiness to define oneself and others with respect to a group identity in the future (Bliuc *et al*., 2007). Indeed, the bipartite attitude networks we derive easily capture the presence of pre‐existing political party membership with socio‐economic attitude data.

## Conclusion

Factional opinion coordination is dynamic and unpredictable yet it can have grave consequences for society. During a pandemic, when many must act collectively to protect the vulnerable few, it is important to maintain non‐partisan solidarity in public health attitudes. We have presented a novel means of detecting factional attitude alignment, and the possible genesis of opposing opinion‐based groups, that could inform ways to inoculate public health messages against partisan interpretations.

## Authors' contributions

Paul Maher (Conceptualization; Data curation; Formal analysis; Methodology; Writing – original draft); Pádraig MacCarron (Conceptualization; Formal analysis; Validation; Visualization; Writing – review & editing); Michael Quayle (Conceptualization; Data curation; Funding acquisition; Methodology; Project administration; Resources; Supervision; Writing – review & editing).

## Conflict of interest

All authors declare no conflict of interest.

## Data Availability

The data that support the findings of this study are available on request from the corresponding author.
